# Anion and Cation Permeability of the Mouse TMEM16F Calcium-Activated Channel

**DOI:** 10.3390/ijms22168578

**Published:** 2021-08-09

**Authors:** Stefano Stabilini, Anna Menini, Simone Pifferi

**Affiliations:** 1 Neurobiology Group, SISSA, International School for Advanced Studies, 34136 Trieste, Italy; stabilini.stefano@gmail.com (S.S.); menini@sissa.it (A.M.); 2 Department of Experimental and Clinical Medicine, Università Politecnica delle Marche, 60126 Ancona, Italy

**Keywords:** ion channel, electrophysiology, permeability

## Abstract

TMEM16F is involved in several physiological processes, such as blood coagulation, bone development and virus infections. This protein acts both as a Ca^2+^-dependent phospholipid scramblase and a Ca^2+^-activated ion channel but several studies have reported conflicting results about the ion selectivity of the TMEM16F-mediated current. Here, we have performed a detailed side-by-side comparison of the ion selectivity of TMEM16F using the whole-cell and inside-out excised patch configurations to directly compare the results. In inside-out configuration, Ca^2+^-dependent activation was fast and the TMEM16F-mediated current was activated in a few milliseconds, while in whole-cell recordings full activation required several minutes. We determined the relative permeability between Na^+^ and Cl¯ (P_Na_/P_Cl_) using the dilution method in both configurations. The TMEM16F-mediated current was highly nonselective, but there were differences depending on the configuration of the recordings. In whole-cell recordings, P_Na_/P_Cl_ was approximately 0.5, indicating a slight preference for Cl¯ permeation. In contrast, in inside-out experiments the TMEM16F channel showed a higher permeability for Na^+^ with P_Na_/P_Cl_ reaching 3.7. Our results demonstrate that the time dependence of Ca^2+^ activation and the ion selectivity of TMEM16F depend on the recording configuration.

## 1. Introduction

The TMEM16 (also named anoctamin or ANO) family is a heterogeneous functional group of ten transmembrane proteins [1,2,3,4]. The first two members, TMEM16A (ANO1) and TMEM16B (ANO2), encode for Ca^2+^-activated Cl¯ channels [5,6,7,8,9] and are involved in several relevant physiological functions, such as control of fluid secretion, modulation of smooth muscle cell excitability, regulation of neuronal signalling in various brain regions and in olfactory and vomeronasal sensory neurons [1,10,11,12,13,14]. TMEM16F (ANO6) and most of the other members of the TMEM16 family, with the exception of TMEM16H (ANO8), function as both Ca^2+^-activated lipid scramblases and ion channels [15,16].

Scramblases mediate the diffusion of phospholipids between the two leaflets of the membrane following their chemical gradient. For example, the activation of scramblases allows phosphatidylserine, that is normally localised in the inner leaflet of the membrane, to be exposed to the cell surface, generating a signal for several physiological processes, such as phagocytosis of apoptotic cells, blood coagulation and bone mineralisation [17]. Suzuki et al., (2010) first showed that TMEM16F is a Ca^2+^-activated phospholipid scramblase and that loss-of-function TMEM16F mutations cause the Scott syndrome, a human bleeding disorder caused by a defect in phospholipid scrambling [16,18]. TMEM16F is also involved in bone development [19], microglial response to injury [20] and virus infections [21,22].

TMEM16F, besides being a scramblase, also mediates Ca^2+^-activated ion channel activity [23] but several discrepancies have been reported regarding the properties of the TMEM16F-mediated current, especially about ion selectivity [24,25]. Indeed, although all reports agree that TMEM16F is a poorly selective channel, several studies performed in whole-cell recordings from TMEM16F heterologously expressed in HEK-293 cells reported a slightly higher permeability to Cl¯ than to Na^+^ [26,27,28,29,30,31,32,33]. On the contrary, recordings using inside-out excised patches showed that TMEM16F was more permeable to cations than to anions [23,34,35]. The TMEM16F Q559K mutant was shown to have a reduced permeability ratio P_Na_/P_Cl_ compared to TMEM16F wild type (wt) when measured in inside-out patches [23], while its ion selectivity in the whole-cell configuration has not been reported yet. Furthermore, a recent study suggested that TMEM16F has a dynamic ion selectivity that depends on Ca^2+^ concentration [36].

As most studies of ion selectivity were obtained by different laboratories using one of the two patch-clamp configurations with different Ca^2+^ concentrations and ionic conditions, in this study we asked whether there is a difference in ion selectivity of the TMEM16F channel in different recording configurations. We performed a side-by-side comparison of the electrophysiological properties of the TMEM16F wt and Q559K mutant using both whole-cell and inside-out configurations with the same ionic solutions. We found that both the time dependence of Ca^2+^ activation and ion selectivity depend on the recording configuration.

## 2. Results

### 2.1. Ca^2+^Activation of the TMEM16F wt Current in Whole-Cell and Inside-Out Configurations

**Figure 1:**
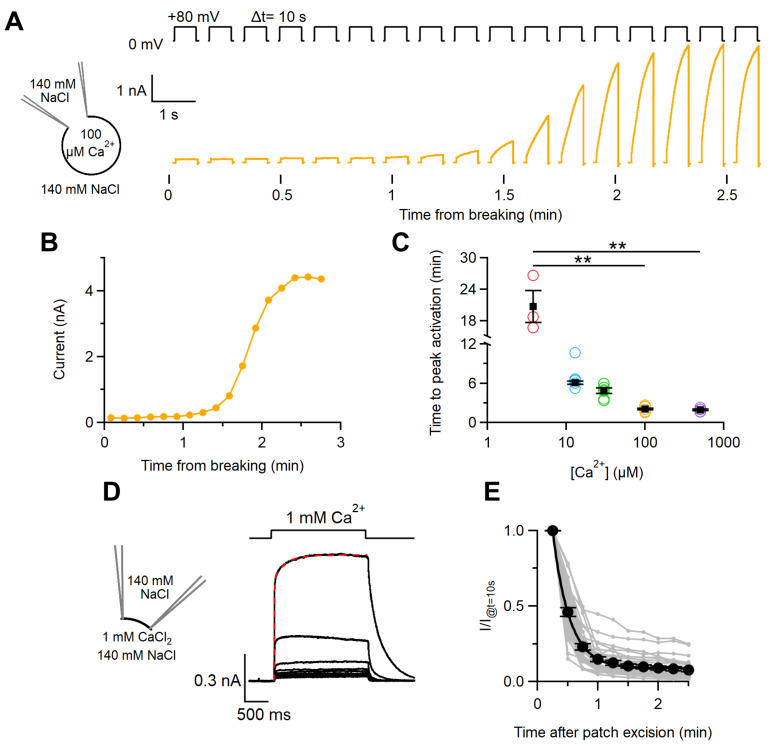
Kinetics of Ca^2+^-dependent activation of the TMEM16F wt-mediated current. (**A**) Whole-cell recordings obtained from TMEM16F wt-transfected cells with a pipette solution containing 100 μM Ca^2+^. The development of the current with time was monitored by repeatedly applying a +80 mV step every 10 s, as indicated in the upper traces. (**B**) Current amplitudes at the end of voltage steps were plotted against the time after membrane breaking for the recordings in (**A**). (**C**) The time required to obtain the maximal current activation was plotted against the intracellular Ca^2+^ concentration (n = 3–8, ** *p* < 0.01 Dunn–Holland–Wolf test after Kruskal–Wallis test). (**D**) An inside-out excised membrane patch expressing TMEM16F wt was repeatedly stimulated every 15 s with a solution containing 1 mM Ca^2+^ as indicated in the upper trace. The holding potential was +60 mV. The red line superimposed to the upper trace is the fit with a double exponential function. (**E**) Ratios between the peak current values measured at various times after patch excision and the value measured after 10 s were plotted against the time after patch excision for several patches (grey dots and lines). Black circles are the average ± sem from different patches (n = 31). The black line is the best fit to a single exponential.

We compared the Ca^2+^-dependent activation of TMEM16F wt currents between whole-cell and inside-out patch-clamp recordings. Figure 1A shows a representative recording in whole-cell configuration obtained by dialyzing HEK-293 TMEM16F wt-expressing cells with a pipette solution containing 100 µM Ca^2+^ and recording the current induced by repeated voltage steps to +80 mV applied every 10 s from the holding potential of 0 mV. The current amplitude gradually increased with time after membrane breaking, reaching a steady state level after approximately 2.5 min (Figure 1B). Recordings with intracellular free Ca^2+^ concentrations varying from 3.8 to 500 µM showed that the time necessary to activate the TMEM16F wt current significantly increased with lower intracellular Ca^2+^, ranging from about 2.5 min at 100 and 500 µM Ca^2+^ to about 20 min at 3.8 µM Ca^2+^ (Figure 1C). Control experiments with HEK-293 transfected with TMEM16A or TMEM16B did not show a significant delay between membrane breaking and current activation, ruling out a possible role of slow Ca^2+^ diffusion in the delayed current activation (Figure S1).

In inside-out patches, we measured the Ca^2+^-dependent activation by using a perfusion system that allows a fast change of solution in less than 10 ms [8]. Figure 1D shows currents activated by the exposure of the cytoplasmic side of a patch to 1 mM Ca^2+^ for 2 s at the holding potential of +50 mV. Unlike the results obtained in whole-cell recordings with free Ca^2+^ in the patch pipette, the TMEM16F current was rapidly activated upon application of 1 mM Ca^2+^ and the time course of Ca^2+^-dependent activation was well fitted with the double-exponential function with time constants of 15 ± 2 ms and 105 ± 31 ms (n = 19). Moreover, the TMEM16F wt current underwent an irreversible decrease in amplitude with time, a process that we define as rundown (Figure 1D,E). To determine the time course of the rundown, we repeatedly exposed the excised inside-out patches to 1 mM Ca^2+^ for 2 s every 15 s, keeping them in the nominally 0 Ca^2+^ solution between Ca^2+^ applications. Subsequent exposures to Ca^2+^ produced currents of decreasing amplitudes (Figure 1D,E). We measured the peak current after each Ca^2+^ application and calculated the ratio between the values obtained at various times after patch excision and the maximal current measured 10 s after patch excision. The rundown was fast in the first minute after patch excision and then slowed down with time and the current sometimes reached a stationary current level. After 2.5 min, the average TMEM16F wt current decreased to 7 ± 1% (*n* = 31) of the starting value (Figure 1E). In contrast, no significant rundown was observed in whole-cell recordings after reaching the steady state level (Figure S2).

**Figure 2:**
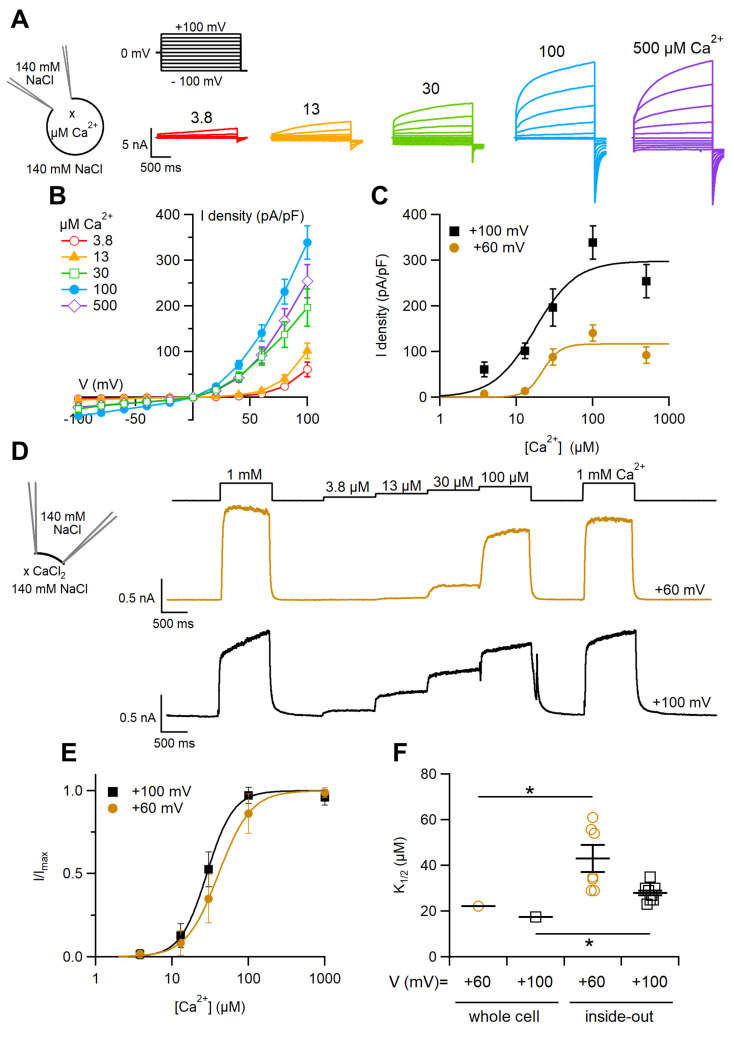
Ca^2+^ sensitivity of the TMEM16F wt-mediated current. (**A**) Representative whole-cell currents recorded from TMEM16F wt-expressing cells with a pipette solution containing the indicated intracellular Ca^2+^ concentrations (x). The voltage protocol is shown at the top of the panel. (**B**) Average steady-state IV relationships from several cells at the indicated Ca^2+^ concentrations (n = 4–10). (**C**) Averages ± sem of the current density at different voltages were plotted against Ca^2+^ concentration (n = 4–10). The continuous lines are the fit with the Hill equation. (**D**) An inside-out excised membrane patch expressing TMEM16F wt was kept in 0 Ca^2+^ and exposed for 1 s to solutions containing different free Ca^2+^ concentrations (x) at the time indicated in the upper traces. The holding potential was +60 mV, or +100 mV as indicated. (**E**) Dose–response relations of activation by Ca^2+^ obtained by normalised currents and fitted to the Hill equation. Current amplitudes were calculated as the average value in the last 50 ms to each Ca^2+^ exposure and the current amplitude in 0 Ca^2+^ was subtracted. (**F**) Comparison of K_1/2_ obtained in different recording configurations (n = 7–8 for inside out, * *p* < 0.05).

To determine the Ca^2+^ sensitivity of the TMEM16F wt-mediated current in whole-cell configuration, we performed experiments dialyzing various cells with intracellular solutions containing different free Ca^2+^ concentrations. Figure 2A shows currents activated by voltage steps of 2.5 s from −100 mV to +100 mV in 20 mV intervals, given from a holding potential of 0 mV. Currents were measured at the time they reached the maximal activation as shown in Figure 1A–C. Unlike the results obtained with TMEM16A and TMEM16B [5,6,7,8,9,37,38], we did not observe a significant change in the rectification of TMEM16F wt depending on the intracellular Ca^2+^, but we measured a strong outward rectification at every Ca^2+^ concentration (Figure 2B). Indeed, the ratio between the current measured at the end of voltage pulses at +100 and −100 mV was 22 ± 8 (n = 6) in the presence of 3.8 µM Ca^2+^ and 20 ± 13 with 100 μM Ca^2+^ (n = 7, *p* = 0.28 Wilcoxon–Mann–Whitney test). To analyse the Ca^2+^-dependence of TMEM16F wt activation at various voltages, we plotted the average current density measured at the end of voltage pulse versus Ca^2+^ concentration and fitted the data with the Hill equation D = D_max_ [c^n^_H_/(c^n^_H_ + *K*_1/2_^n^_H_)], where D is the current density, D_max_ is the maximal current density, c is the Ca^2+^ concentration, *K*_1/2_ is the Ca^2+^ concentration producing half-maximal current activation and n_H_ is the Hill coefficient. K_1/2_ slightly decreased with membrane depolarisation from 22.2 μM at +60 mV to 17.4 μM at +100 mV, while the Hill coefficient ranged from 1.6 to 3.8 (Figure 2C,F).

In inside-out patches, we measured the dependence of the TMEM16F wt-induced current on the intracellular Ca^2+^ concentration by exposing every single patch to solutions containing various Ca^2+^ concentrations. Each patch was kept in nominally 0 Ca^2+^ solution, exposed for 1 s to 1 mM Ca^2+^ and returned to 0 Ca^2+^ for 1 s to make sure that all Ca^2+^ was removed. Then the patch was exposed to increasing Ca^2+^ concentrations in the range between 3.8 and 100 μM and returned to 0. Finally, a test with 1 mM Ca^2+^ was repeated to establish if there was rundown of the current. Experiments were performed just after patch excision and currents measured at different Ca^2+^ concentrations were normalised to the average value obtained with 1 mM Ca^2+^ before and after the application of various test Ca^2+^ concentrations to minimise the possible effect of rundown. Figure 2D illustrates the results of representative dose-response experiments at the holding potentials of +60 mV and +100 mV. Normalised currents versus the Ca^2+^ concentration are plotted in Figure 2E and fitted by the Hill equation: I/I_max_ = c^nH^/(c^nH^ + *K*_1/2_^nH^), where c is the Ca^2+^ concentration, *K*_1/2_ is the Ca^2+^ concentration producing half-maximal current activation and n_H_ is the Hill coefficient. Similar to the data obtained in whole cell recordings, K_1/2_ slightly decreased with membrane depolarisation from 43 ± 6 μM at +60 mV to 28 ± 1 μM at +100 mV (n = 7–8; *p* = 0.031 unpaired *t*-test, Figure 2F), while the Hill coefficient ranged from 2.4 to 2.8. Although K_1/2_ values measured in inside-out were significantly higher than those obtained from whole-cell recordings, they all ranged between 17.4 and 43 μM, indicating a low apparent affinity of TMEM16F wt for Ca^2+^ in both configurations.

### 2.2. Comparison between Ion Selectivity of TMEM16F wt in Whole-Cell and Inside-Out Configurations

**Figure 3:**
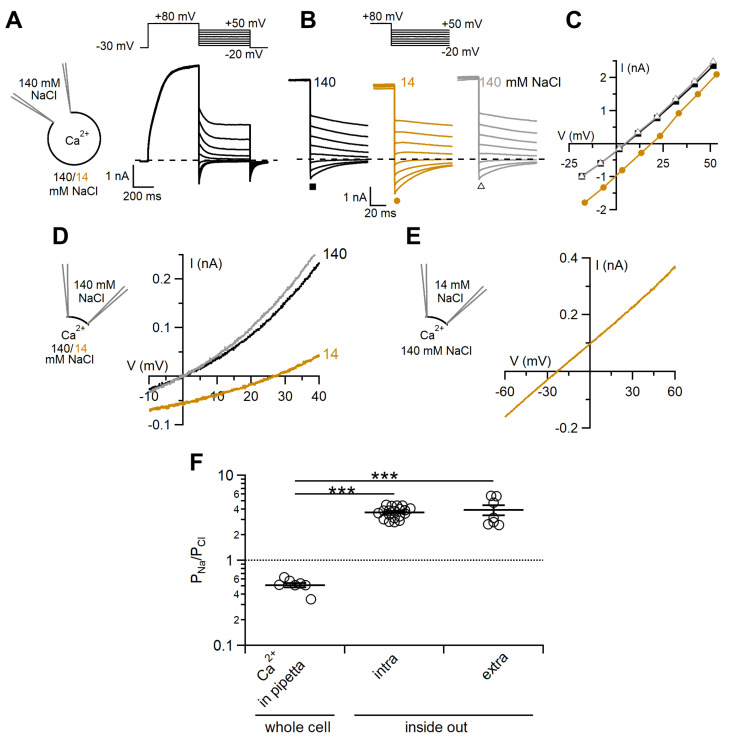
TMEM16F wt ion permeability ratio P_Na_/P_Cl_ depends on the recording configuration. (**A**,**B**) Representative whole-cell voltage-clamp recordings obtained from representative TMEM16F wt-transfected cells with intracellular solution containing 50 μM Ca^2+^. Voltage protocols are shown at the top of the panels. (**B**) A cell was first exposed to a control solution containing 140 mM NaCl, then to 14 mM NaCl and returned to 140 mM NaCl. (**C**) IV relations measured from tail currents of the cell shown on the left with 140 mM NaCl (squares), 14 mM NaCl (circles) and after returning to 140 mM NaCl (triangles). (**D**,**E**) Inside-out patches expressing TMEM16F wt were exposed to the indicated NaCl concentrations and the IV relations were determined by voltage ramps from +100 to −80 mV. Only the regions around the reversal potentials are shown. Currents were activated by 100 μM CaCl_2_ and leakage currents measured in 0 Ca^2+^ were subtracted. The pipette solution contained 140 mM NaCl (D) or 14 mM NaCl (**E**). (**F**) Comparison of P_Na_/P_Cl_ calculated with the Goldman-Hodgkin-Katz equation from reversal potentials measured in different recording conditions (n = 7–19; *** *p* < 0.001 Tukey test after ANOVA, F = 48.256 *p* = 4.2 × 10^−10^).

To compare the ion selectivity of TMEM16F wt in the whole-cell and inside-out configurations we first investigated the permeability ratio between Na^+^ and Cl¯ by diluting the external NaCl concentration from 140 mM to 14 mM. As the I–V relations measured in whole-cell recordings showed a very pronounced outward rectification, we measured tail currents by first activating the channels with a prepulse at +80 mV followed by voltage steps between −20 and +50 mV (Figure 3A,B). It is interesting to note that the I–V relation obtained from tail currents measured at the beginning of each step was almost linear (Figure 3C and Figure 5A), whereas the steady state I–V had a strong outward rectification (Figure 2B), clearly demonstrating that the I–V relation of the open channel was almost linear, and therefore the strong outward rectification mainly depends on voltage-dependent gating.

In whole-cell recordings, dilution of NaCl in the extracellular solution caused a positive shift of reversal potential to 14 ± 1 mV as expected for channels more permeable to anions than to cations (Figure 3C). The relative permeability ratio between Na^+^ and Cl¯ (P_Na_/P_Cl_) calculated with the Goldman–Hodgkin–Katz equation was 0.52 ± 0.03 (n = 7, Figure 3F), indicating that the TMEM16F wt channel was more permeable to Cl¯ than to Na^+^. Similar results were obtained also using voltage ramps (Figure S3).

In inside-out patches, reduction of the cytoplasmic NaCl concentration from 140 to 14 mM caused a positive shift of the reversal potential to 25.5 ± 0.6 mV (n = 19), as expected for channels more permeable to cations than to anions. P_Na_/P_Cl_ was 3.7 ± 0.1 (n = 19), very different from the value of 0.52 ± 0.03 (n = 7) calculated from whole-cell recordings (Figure 3F). Thus, the TMEM16F wt channel recorded in inside-out excised patches was more permeable to Na^+^ than to Cl¯, whereas it was the opposite in the whole-cell configuration. However, since NaCl concentrations were modified at different sides of the channel in the two patch-clamp configurations (Figure 3C,F), we reasoned that the different permeabilities could be due to an asymmetric channel pore. To test this possibility, we evaluated the ionic permeability in the inside-out configuration by reducing the NaCl concentration to 14 mM in the pipette solution bathing the extracellular side of the membrane (Figure 3E). The average reversal potential in these ionic conditions was -26 ± 2 mV (n = 7) and P_Na_/P_Cl_ was 3.9 ± 0.5 (n = 7), showing that also in these experimental conditions Na^+^ was more permeant than Cl¯. Thus, the significant difference between P_Na_/P_Cl_ measured in whole-cell or in the inside-out configuration was not due to an asymmetric channel pore (Figure 3F).

**Figure 4:**
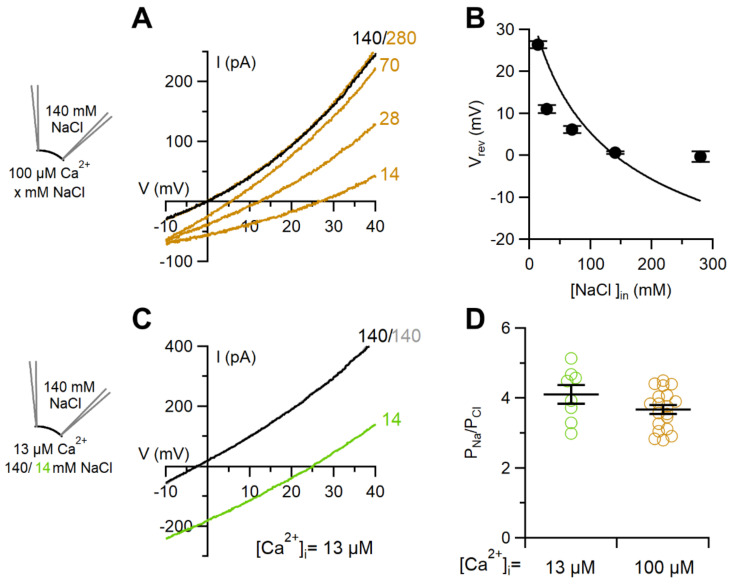
TMEM16F wt ion permeability ratio P_Na_/P_Cl_ in excised inside-out patches depends on ionic strength but not on intracellular Ca^2+^ concentration. (**A**) An inside-out patch expressing TMEM16F wt was exposed to the indicated cytoplasmatic NaCl concentrations (in mM) and the IV relations were determined by a voltage ramp from +100 to −80 mV. Only the regions around the reversal potentials are shown. The current was activated by 100 μM CaCl_2_. Leakage currents measured in 0 Ca^2+^ were subtracted. (**B**) Average reversal potentials (V_rev_) corrected for liquid junction potentials were plotted versus NaCl concentrations (n = 11–19). The continuous line was calculated according to the Goldman-Hodgkin-Katz equation with P_Na_/P_Cl_ = 3.7. (**C**) An inside-out patch expressing TMEM16F was exposed to 140 or 14 mM cytoplasmatic NaCl and the IV relation was determined by a voltage ramp from +100 to −80 mV. The current was activated by 13 μM CaCl_2_. (**D**) Comparison of P_Na_/P_Cl_ calculated with the Goldman-Hodgkin-Katz equation from reversal potentials measured with the indicated Ca^2+^ concentration (n = 8–19, *p* = 0.19 unpaired *t*-test).

Since using the dilution method could cause significant deviations from the expected value of P_Na_/P_Cl_ from the Goldman–Hodgkin–Katz equation [36,39], we tested this possibility in inside-out patches by varying the NaCl concentration in the bath solution from 14 mM to 280 mM (Figure 4A) and we found significant deviations from prediction of the Goldman–Hodgkin–Katz equation (Figure 4B). Indeed, P_Na_/P_Cl_ obtained with solutions containing 280 and 70 mM NaCl were 1.98 ± 0.09 and 2.3 ± 0.2 respectively, significantly different from the value of 3.7 ± 0.1 measured in 14 mM NaCl (n = 12–19; *p* < 0.001 Dunn–Holland–Wolf test after Kruskal–Wallis test *p* = 2 × 10^−6^). However, although P_Na_/P_Cl_ changed with the variation of ionic strength, the TMEM16F wt channel in inside-out patches remained more permeable to Na^+^ than to Cl¯.

Since it has been recently reported that P_Na_/P_Cl_ of TMEM16F wt in inside-out patches may depend on Ca^2+^ concentration [36], we investigated this possibility by reducing Ca^2+^ from 100 to 13 µM, a concentration that produced about 10% of the maximal current at +100 mV (Figure 2E). We found that the value of P_Na_/P_Cl_ measured by diluting the bath solution from 140 to 14 mM NaCl in 13 µM Ca^2+^ (Figure 4C) was not significantly different from that determined in 100 µM Ca^2+^ (Figure 4D), indicating that P_Na_/P_Cl_ permeability of TMEM16F wt in inside-out patches did not change with Ca^2+^ concentration. Furthermore, in patches with a slow rundown, we repeated measurements of the reversal potential at different times after patch excision and found similar values, indicating that the ion selectivity did not change with time.

**Figure 5:**
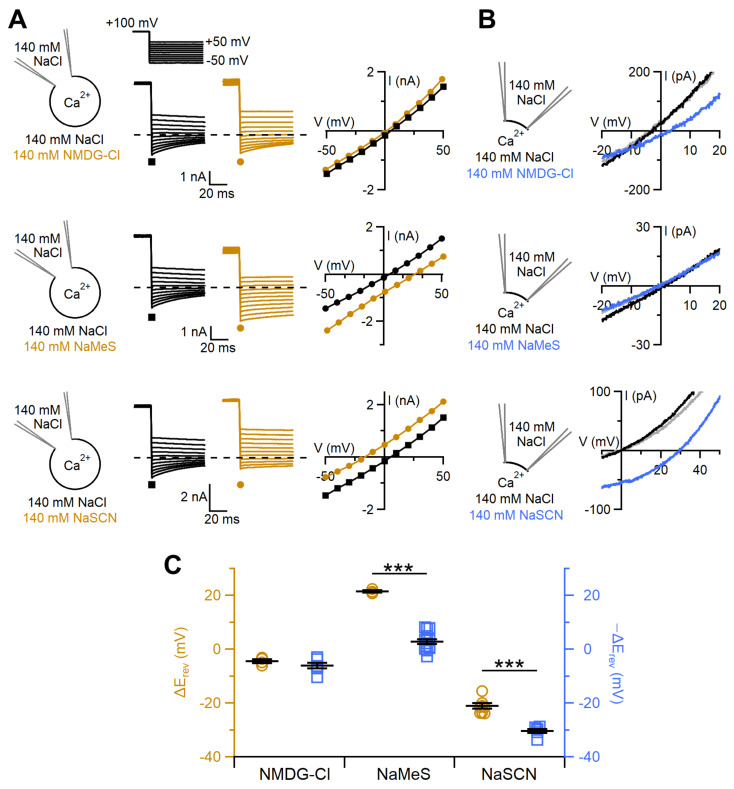
TMEM16F wt ion permeability depends on the recording configuration. (**A**) Representative whole-cell voltage-clamp recordings obtained from TMEM16F wt-transfected cells with intracellular solution containing 100 μM CaCl_2_. Each cell was exposed to a control solution containing 140 mM NaCl (black traces) or other salts as indicated (brown traces). On the right, IV relations measured from tail currents of the cells on the left with 140 mM NaCl (black squares) or 140 mM of the indicated salt (brown circles). (**B**) An inside-out patch expressing TMEM16F wt was exposed to bath solutions containing 140 mM NaCl (black traces) or the indicated salts (blue traces). The grey traces represent the wash out with NaCl. IV relations were determined by voltage ramps from +100 to −80 mV. Only the regions around the reversal potentials are shown. Currents were activated by 1 mM CaCl_2_ and leakage currents measured in 0 Ca^2+^ were subtracted. (**C**) Comparison of the shift of reversal potentials obtained in different recording configurations for the indicated ionic solutions (*** *p* < 0.001, for NaMeS: *p* = 9.7 × 10^−12^ and for NaSCN: *p* = 8 × 10^−6^, unpaired *t*-test).

To further investigate the permeability of TMEM16F wt to different cations and anions, we replaced NaCl in the bathing solution with NMDG-Cl, NaMeS or NaSCN and compared the shift of the reversal potentials by taking into account that ion replacements occurred at the opposite side of the membrane in the two patch-clamp configurations (Figure 5A,B). We found that the large cation NMDG^+^ was poorly permeant through the TMEM16F wt pore without any significant difference between whole-cell and inside-out recordings, indeed the change of reversal potential was −2.9 ± 1 mV in whole cell and 4.3 ± 0.7 mV in inside-out patches (Figure 5C, n = 4–5, *p* = 0.29, unpaired *t*-test). The large anion MeS¯ was also permeant, although at different extents depending on the recording method. Indeed, substitution of NaCl with NaMeS in whole-cell shifted the reversal potential of 21.5 ± 0.5 mV (n = 3), indicating that MeS¯ was much less permeant than Cl¯, whereas in inside-out the change was only −1.6 ± 0.7 mV (n = 14) showing a very small difference between MeS¯ and Cl¯ permeability (Figure 5C). SCN¯ showed a higher permeability than Cl¯ in both recording conditions, although it was found to be more permeant in the inside-out configuration (Figure 5C). Using the inside-out configuration, we also tested the permeation of several other anions finding the following permeability sequence for TMEM16F wt: SCN¯ > I¯ > NO_3_¯ > Br¯ > Cl¯ (Figure S4).

### 2.3. Comparison between Ion Selectivity of TMEM16F Q559K in Whole-Cell and Inside-Out Configurations

TMEM16F Q559K is considered to be a pore mutant because it altered ion selectivity in inside-out patches by reducing the selectivity of the channel to Na^+^ with respect to Cl¯ [23]. We repeated the same experiments in excised patches and investigated whether a change of selectivity was present also in whole-cell recordings.

First, we characterised the activation by Ca^2+^ of the Q559K mutant that showed, as the TMEM16F wt, a slow increase of current with time in whole-cell and a fast activation in the inside-out configurations (Figure S5). The dose-response curve in inside-out patches showed a lower apparent Ca^2+^ sensitivity for Q559K compared to wt.

**Figure 6:**
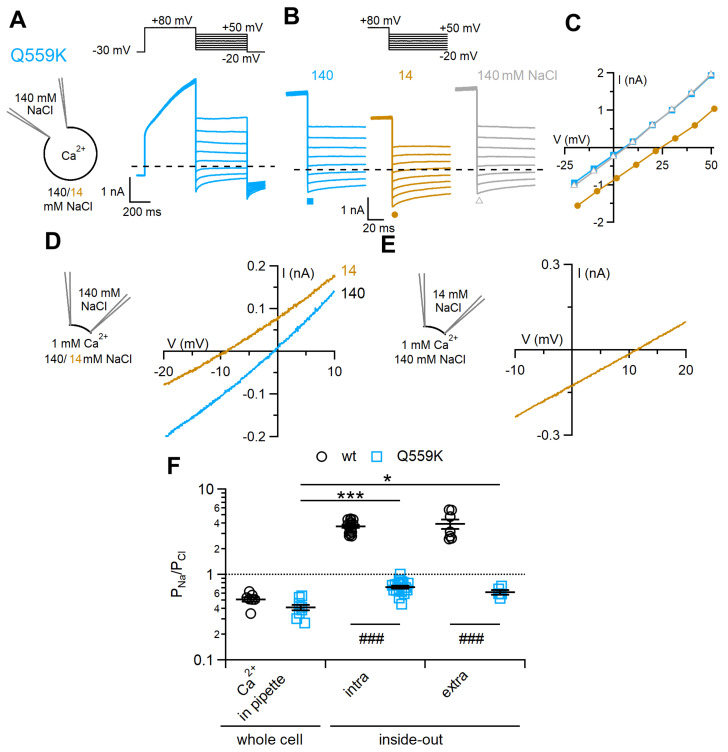
Q559K ion permeability ratio P_Na_/P_Cl_ depends on the recording configuration. (**A**,**B**) Representative whole-cell voltage-clamp recordings obtained from TMEM16F Q559K mutant-transfected cells with intracellular solution containing 300 μM Ca^2+^. Voltage protocols are shown at the top of the panels. (**B**) A cell was exposed to a control solution containing 140 mM NaCl, then to 14 mM NaCl, followed by wash out with 140 mM NaCl. (**C**) IV relations measured from tail currents of the cell shown on the left with 140 mM NaCl (squares), 14 mM NaCl (circles) and after wash out (triangles). (**D**,**E**) Inside-out patches expressing TMEM16F were exposed to the indicated NaCl concentrations, and the IV relations were determined by voltage ramps from +100 to −80 mV. Only the regions around the reversal potentials are shown. Currents were activated by 1 mM CaCl_2_ and leakage currents measured in 0 Ca^2+^ were subtracted. The pipette solution contained 140 mM NaCl (**C**) or 14 mM NaCl (**D**). (**F**) Comparison of P_Na_/P_Cl_ in different recording conditions for Q559K and wt (n = 5–19; *** *p* < 0.001, * *p* < 0.05 Tukey test after ANOVA, F = 18.271, *p* = 8.36 × 10^−6^; ### *p* < 0.001, unpaired *t*-test).

For ion selectivity, we confirmed the previous data in inside-out patches showing that Q559K was less permeable to Na^+^ than TMEM16F wt. In 1 mM Ca^2+^, P_Na_/P_Cl_ was 0.71 ± 0.01 (n = 18) in Q559K (Figure 6D,F) to be compared with the value of 3.7 ± 0.1 (n = 19) in TMEM16F wt. Moreover, reduction from 140 to 14 mM NaCl in the pipette solutions bathing the extracellular side of the membrane did not significantly modify P_Na_/P_Cl_ that had a value of 0.62 ± 0.04 (n = 5), indicating that the pore of the Q559K mutant was not asymmetric, similarly to the wt pore (Figure 6E,F). In addition, we also confirmed that ion selectivity of Q559K depends on intracellular Ca^2+^ concentration as previously reported [36]. Indeed, we found that activation of the Q559K channel by 13 µM Ca^2+^ produced a significantly higher P_Na_/P_Cl_ of 1.9 ± 0.1 compared to 0.71 ± 0.01 measured in 1 mM Ca^2+^ (Figure S6), although we did not measure any change of P_Na_/P_Cl_ with Ca^2+^ concentration in the TMEM16F wt channel (Figure 4D).

Whole-cell recordings showed that the value of P_Na_/P_Cl_ for the Q559K mutant activated by 300 µM Ca^2+^ was 0.41 ± 0.03 (n = 8, Figure 6A–C,F), significantly different from 0.62 ± 0.04 measured for Q559K in inside-out, but not significantly different from 0.52 ± 0.03 (n = 7) for the wt channel (Figure 6F), showing that the mutation did not modify selectivity in the whole-cell configuration.

### 2.4. Ion Selectivity and Scramblase Activity

**Figure 7:**
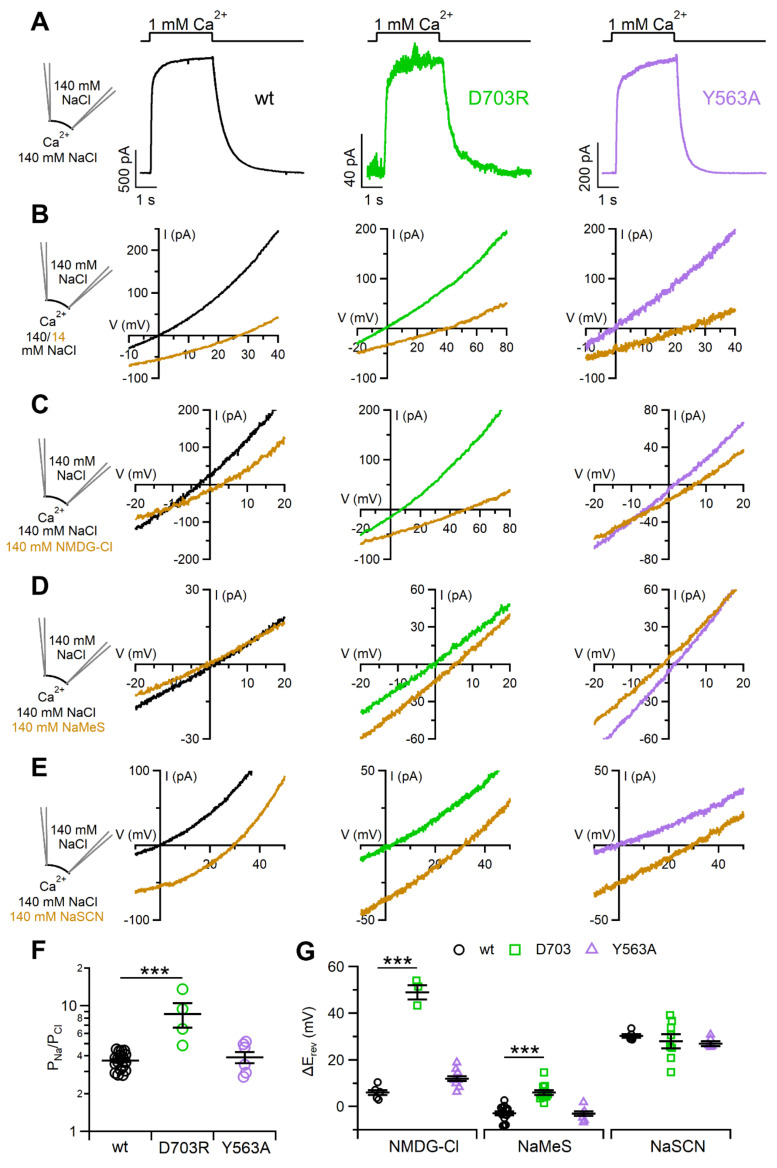
Ion permeability of the TMEM16F wt, D703R and Y563A mutants. (**A**) Inside-out excised membrane patches expressing TMEM16F wt or the indicated mutant were stimulated with a solution containing 1 mM Ca^2+^ at the time indicated in the upper traces. The holding potential was +60 mV. Patches were exposed to 140 mM NaCl and (**B**) 14 mM NaCl, (**C**) 140 mM NMDG-Cl, (**D**) 140 mM NaMeS or (**E**) 140 mM NaSCN. IV relations were determined by voltage ramps from +100 to −80 mV. Only the regions around reversal potentials are shown. Leakage currents measured in 0 Ca^2+^ were subtracted. (**F**) Comparison of P_Na_/P_Cl_ for TMEM16F wt and the indicated mutants (n = 3–19, *** *p* < 0.001, Dunnett test after ANOVA F = 19.303, *p* = 7.26 × 10^−6^). (**G**) Comparison of the shift of reversal potentials after the replacement of 140 mM NaCl with 140 mM NMDG-Cl, NaMeS or NaSCN for TMEM16F wt and the indicated mutants (for NMDG-Cl: *** *p* < 0.001, Dunnett test after ANOVA F = 30.569, *p* = 7.79 × 10^−6^; for NaMeS: *** *p* < 0.001, Dunnett test after ANOVA F = 26.3, *p* = 3.86 × 10^−7^; for NaSCN: ANOVA, F = 1.499, *p* = 0.25).

As TMEM16F functions both as Ca^2+^-activated ion channel and as Ca^2+^-activated lipid scramblase, we also tested if ion selectivity depends on scramblase activity by using two mutants that differently affect the scrambling activity, the scrambling-deficient D703R mutant and the constitutive scrambling Y563A mutant [40]. Figure 7A shows currents activated by the exposure of the cytoplasmic side of inside-out excised patches to 1 mM Ca^2+^ at the holding potential of +60 mV. Currents were rapidly activated by Ca^2+^ in the two mutants with kinetics similar to the wt channel. To determine if scramblase activity modifies the P_Na_/P_Cl_ permeability, we measured reversal potentials upon reduction of bath NaCl from 140 to 14 mM (Figure 7B). The positive shifts of reversal potentials, 38 ± 3 mV (n = 3) for D703R and 26 ± 2 mV (n = 6) for Y563A, indicate that both mutants measured in the inside-out configuration were more selective for Na^+^ than for Cl¯ similarly to wt (25.5 ± 0.6 mV, n = 19). Furthermore, P_Na_/P_Cl_ (3.9 ± 0.4, n = 6) for the constitutive scramblase mutant Y563A was not significantly different from the wt value, whereas P_Na_/P_Cl_ for the scrambling-deficient D703R mutant (9 ± 2, n = 3) was significantly higher than the wt value (3.7 ± 0.1, Figure 7F), indicating an even higher selectivity for Na^+^ than for Cl¯ of D703R compared to wt.

Furthermore, we extended the comparison of permeabilities to other ions by substituting NaCl in the bathing solution with NMDG-Cl, NaMeS or NaSCN (Figure 7C–E,G). All three channels were permeable to the large cation NMDG^+^ and to the large anion MeS¯, with the scrambling-deficient D703R mutant more permeable to these ions than the other two channels. SCN¯ was equally permeant in the three channels. Thus, data obtained with the constitutive scrambling Y563A mutant were not significantly different from the wt, while the scrambling-deficient D703R mutant showed some difference (Figure 7G).

## 3. Discussion

Our results provide a detailed side-by-side comparison of the electrophysiological properties of TMEM16F wt and Q559K recorded in the whole-cell and inside-out modes. We measured the important differences between several features of the TMEM16F-mediated currents depending on the recording configuration. Indeed, in inside-out recordings, the TMEM16F wt channel was rapidly activated by Ca^2+^ and had a higher permeability to Na^+^ than to Cl¯, while in the whole-cell configuration the channel was activated by Ca^2+^ only after a delay of several minutes and was slightly more permeable to Cl¯ than to Na^+^.

### 3.1. Calcium Activation

Our data confirm that the kinetics of activation by Ca^2+^ strongly depend on the recording configuration. Indeed, in the inside-out configuration, both TMEM16F wt and Q559K currents were rapidly activated within a few ms from Ca^2+^ application (Figure 1D) in agreement with previous results [23,35,36]. Moreover, the amplitude of TMEM16F Ca^2+^-activated currents in inside-out underwent a progressive rundown as previously observed by Ye et al. [41], who showed that the rundown was due to depletion of phosphatidylinositol-(4, 5)-bisphosphate (PIP_2_) in excised patches. Both the fast Ca^2+^ activation and the rundown in inside-out patches resemble those measured in TMEM16A [42] and TMEM16B [8,9].

In contrast, the TMEM16F Ca^2+^-activated current in whole-cell recordings slowly and progressively developed after a delay of some minutes following membrane breaking and did not show rundown. The time necessary to obtain the maximal current was about 2 min with 100 μM Ca^2+^ in the patch pipette and increased to about 20 min with a reduction in intracellular Ca^2+^ to 3.8 μM (Figure 1B,C). This slow activation is in agreement with the results from previous studies for TMEM16F [27,43], while it differs from the fast kinetics of activation by Ca^2+^ measured in whole-cell mode for TMEM16A and TMEM16B [5,7,8,38]. For TMEM16E, which acts both as an ion channel and scramblase similarly to TMEM16F, whole-cell currents were rapidly activated by intracellular Ca^2+^ in a study by Di Zanni et al. [44], while another study reported current activation after a delay of several minutes [45]. For TMEM16F, activation kinetics by Ca^2+^ in whole-cell mode have been shown to be modified by the actin cytoskeleton, as molecules that interfere with actin polymerisation accelerated TMEM16F activation [46].

Our dose-response experiments showed that TMEM16F wt requires several μM of Ca^2+^ to be activated and that the Q559K mutant has an even lower Ca^2+^ sensitivity, in agreement with the previous results [34,35]. The low sensitivity of the Q559K mutant to Ca^2+^ is likely to explain why a previous attempt by Scudieri et al. [47] to measure Q559K ion selectivity in whole-cell did not produce any current as they were using a Ca^2+^ concentration of 20 μM that was sufficient to activate the TMEM16F wt current but probably too low to measure a current in the mutant.

The low Ca^2+^ sensitivity of TMEM16F suggests that its activation requires the presence of local nano- or micro-domains where Ca^2+^ can reach elevated local concentrations. A similar organisation was reported also for the activation of Ca^2+^-activated K^+^ channels [48] and is important for the activation of TMEM16B in the cilia of olfactory sensory neurons [12,49].

### 3.2. Ion Selectivity

Measurements of the ion selectivity of TMEM16F wt gave rise to contrasting results in previous reports and our side-by-side comparison indicates that several discrepancies are due to the different recording configurations used. In our experiments, TMEM16F wt measured in the presence of a high Ca^2+^ concentration had a slightly higher permeability to Cl¯ than to Na^+^ in whole-cell, with a permeability ratio P_Na_/P_Cl_ of 0.52, while an opposite higher permeability to Na^+^ than to Cl¯, with P_Na_/P_Cl_ of 3.7, was measured in the inside-out configuration (Figure 3). We also tested the possibility that the different permeabilities could be due to an asymmetric channel pore or to a change of ionic strength, because NaCl concentration was reduced at different sides of the channel in the two patch-clamp configurations (extracellular in whole-cell and intracellular, in inside-out). Reduction of the NaCl concentration from 140 to 14 mM at the extracellular side of the membrane produced a P_Na_/P_Cl_ of 3.9 similar to the value of 3.7 obtained by varying NaCl at the intracellular side, showing that also in these experimental conditions Na^+^ was more permeant than Cl¯ in the inside-out mode. Thus, the significant difference between P_Na_/P_Cl_ measured in whole-cell and in the inside-out configuration was not due to an asymmetric channel pore or to a change of ionic strength (Figure 3E). Moreover, as a previous study [36] reported that permeability may change with Ca^2+^ concentration, we also compared P_Na_/P_Cl_ when TMEM16F wt was activated by a lower Ca^2+^ concentration and found that the value measured at 13 μM (4.1) was not different from that measured at 100 μM Ca^2+^ (3.7, Figure 4D).

TMEM16F wt is permeable to several anions, including the large anion MeS¯, and to many cations, including the large cation NMDG^+^ (Figure 5). The anion permeability sequence in inside-out was SCN¯ > I¯ > NO_3_¯ > Br¯ > Cl¯ (Figure S1) in agreement with other studies in inside out and whole-cell recordings [26,27,35] and is similar to that measured for TMEM16A and TMEM16B [7,8,9,50]. These data show that anions with lower dehydration energy are more permeant compared to those with higher dehydration energy and suggest a conserved mechanism of anion permeation among these channels.

In agreement with a previous report [23], we found that cation selectivity in inside-out patches was reduced by the Q559K pore mutation. In our experiments, the value of P_Na_/P_Cl_ was 0.71 (or 0.62) when NaCl concentration was reduced from 140 to 14 mM at the intracellular (or extracellular) side of the patch membrane and channels were activated by high Ca^2+^ concentrations. P_Na_/P_Cl_ increased to 1.9 when TMEM16F Q559K was activated by 13 μM Ca^2+^ (Figure S3), in agreement with the trend of permeability change with Ca^2+^ concentration previously reported [36].

When the permeability ratio was measured in whole-cell, P_Na_/P_Cl_ of Q559K was 0.41, slightly lower than the value of 0.71 measured in inside-out. Surprisingly, P_Na_/P_Cl_ of the Q559K pore mutant (0.41) measured in whole-cell was not different from the value of TMEM16F wt (0.52), showing that the mutation did not modify selectivity in the whole-cell configuration.

We also investigated whether the Ca^2+^-activated scramblase activity of TMEM16F could play a role in ion selectivity. Indeed, previous work [43] identified a protein domain required for scrambling of TMEM16F that is sufficient to produce scramblase activity also in TMEM16A, and Jiang et al. [51] showed that the TMEM16A V543S mutation conferred scramblase activity to TMEM16A. In both studies, TMEM16A (that is anion selective) became more cation selective after phospholipid scrambling occurred, showing that ion selectivity was influenced by scramblase activity. In another study (Malvezzi et al., 2013), ion channel properties of afTMEM16 (TMEM16 from the fungus *Aspergillus fumigatus*) reconstituted in lipid bilayers were reported to strongly depend on the lipid composition, suggesting that different lipid environments generated by the scramblase activity may affect the properties of the TMEM16F pore. Le et al. [40] identified residues that play important roles in scramblase activity and showed that the TMEM16F Y563A mutant Fproduced a constitutive scrambling activity while the D703R mutant abolished scrambling activity. They also compared ion selectivity between Y563A and wt (but not for D603R) showing that P_Na_/P_Cl_ increased in the mutant (Figure S4E,F in [40]). In our experiments, P_Na_/P_Cl_ for the constitutive scramblase Y563A mutant was 3.9 similar to 3.7 measured in the wt, while it increased to 9 for the scrambling-deficient D703R mutant, indicating that a higher selectivity for Na^+^ than for Cl¯ was measured in the absence of scramblase activity (Figure 7). We also compared the selectivity to other anions and cations and confirmed the absence of differences between Y563A and wt, while the large cation NMDG^+^ and anion MeS¯ were more permeant in the scrambling-deficient D703R mutant than in wt. These results do not support the possibility that TMEM16F could become more cation selective after lipid scrambling as was shown for TMEM16A [43,51].

One possible explanation of the differences we measured in the two configurations could be the different tension of the membrane in inside-out with respect to whole-cell configuration [52]. Indeed, recent studies showed that many ion channels are modulated by mechanical forces applied to lipid bilayer [53,54].

It is also tempting to speculate that the different data obtained using the two recording configurations could be due to a still unidentified factor that binds TMEM16F in the whole-cell configuration and is lost in excised patches. Interestingly, calmodulin has been shown to be able to modulate the Ca^2+^ sensitivity [55] and permeability [56] of TMEM16A, even if these results were challenged [57]. Several other possible binding partners of TMEM16 proteins have been reported. Huang et al. [58] showed that TMEM16C binds the Na^+^-activated K^+^ channel Slack, increasing the single-channel activity and sodium sensitivity of Slack channels; structural data by Pan et al. [59] revealed that TMC1 channels share a common architecture with the TMEM16 channel, raising the possibility that some TMC-binding proteins could also bind TMEM16F; Avalos-Prado et al. [60] reported that KCNE1 is an auxiliary subunit of TMEM16A. However, also several other modulators including cholesterol, fatty acids, phosphorylation, have been shown to regulate the activity of some TMEM16 family members [4,61].

### 3.3. Conclusions

In summary, our data provide a clear demonstration that the time dependence of Ca^2+^ activation and the ion selectivity of TMEM16F depend on the recording configuration, contributing to a clarification of the previous conflicting data.

## 4. Materials and Methods

### 4.1. Cell Culture and Transfection

HEK-293 cells were grown in medium composed of DMEM (Gibco, Milano, Italy) supplemented with 10% foetal bovine serum (Sigma, Milano, Italy), 100 IU/mL penicillin and 100 μg/mL streptomycin (Sigma, Milano, Italy) at 37 °C in a humidified atmosphere of 5% CO_2_.

pEGFP-N1 plasmids containing the cDNA of mouse TMEM16F wt (version 1 as in [46]) or Q559K mutant were provided by Professor Lily Jan (UCSF, San Francisco, CA, USA). pEGFP-N1 plasmids containing the cDNA of D703R and Y563A mutants were provided by Professor Huanghe Yang (Duke University, Durham, NC, USA). HEK-293 cells were transfected with 2 μg TMEM16F cDNA using the transfection reagent XtremeGENE (Roche Diagnostic, Milano, Italy). Twenty-four hours after transfection, the cells were sub-cultured in 35-mm Petri dishes at a lower density. Electrophysiological recordings were performed between 48 and 72 h after transfection.

### 4.2. Electrophysiological Recordings

TMEM16F-transfected HEK-293 cells were identified by EGFP fluorescence using an Olympus IX70 microscope (Olympus, Tokyo, Japan) equipped with the appropriate filter. TMEM16F currents were recorded in whole-cell or inside-out configurations in voltage-clamp mode using an Axopatch 1-D amplifier controlled by Clampex 9.2 via Digidata 1322A (Axon Instruments, San Jose, CA, USA). The data were acquired at a rate of 10 kHz, and the signals were low-pass filtered at 5 kHz. Patch electrodes were made of borosilicate glass (WPI, Friedberg, Germany) and pulled with a PP-830 micropipette puller (Narishige, Tokyo, Japan). Pulled patch electrodes had a resistance of 2–3 MΩ for whole-cell recordings and 0.5–1 MΩ for the inside-out configuration when filled with pipette solution.

The bath was ground with a 3 M KCl agar bridge connected with an Ag/AgCl reference electrode. In experiments with pipette solution without Cl¯, a 3 M KCl agar bridge was used. All experiments were conducted at room temperature. Solutions were rapidly changed close to the cells or excised patches using multibarrel glass tubes. Each tube composing the multibarrel had an internal diameter of 0.9 mm (Vitro Dynamics, Trenton, NJ, USA). Changes between different solutions were performed using the Perfusion Fast-Step SF-77B (Warner Instrument Corp., Holliston, MA, USA).

For IV relations in the inside-out configuration, we exposed the patches to Ca^2+^-containing solution for 1 s at +100 mV and then applied a ramp from +80 to −80 mV at 0.36 mV/ms. Leak currents measured in nominally 0 Ca^2+^ solution were subtracted. The reversal potential (E_rev_) was estimated by using a fit with the polynomial function I = a_1_ + a_2_V + a_3_V^2^ + a_4_V^3^ + a_5_V^4^ + a_6_V^5^ + a_7_V^6^, where I is the current, V is the voltage and a_i_ are numerical parameters.

For whole-cell recordings of tail currents, the protocol consisted of voltage steps of 500 ms duration from a holding potential of 0 mV to + 80 mV followed by voltage steps ranging from −20 to +50 mV. A single-exponential function (I = A_1_ exp (−t/t_1_)), was fitted to tail currents to extrapolate the tail current value at the beginning of the step. Tail current values were plotted as a function of voltage, and E_rev_ was estimated using a fit with a polynomial function.

To measure dose-responses and ion selectivity, currents in whole-cell were measured at the time they reached the maximal activation as shown in Figure 1A–C, while experiments in inside-out were performed just after patch excision to avoid current rundown (Figure 1D,E). However, in patches with a slow rundown several recordings were obtained from the same patch.

Liquid junction potentials were calculated using the pClampex software (Axon Instruments, San Jose, CA, USA), and applied voltages were corrected off-line.

### 4.3. Solutions

Cells were kept in mammalian Ringer’s solution composed of (in mM) 140 NaCl, 5 KCl, 2 CaCl_2_, 1 MgCl_2_, 10 glucose and 10 HEPES, pH 7.4 with NaOH.

For whole-cell recordings, the pipette solution contained (in mM) 140 NaCl, 10 HEPES and 10 HEDTA, pH 7.2 and 5.86, 8.26 or 9.26 mM CaCl_2_ to obtain 3.8, 13 or 30 µM free Ca^2+^, respectively. For intracellular solutions containing Ca^2+^ concentrations >30 µM HEDTA was omitted and an appropriate amount of CaCl_2_ was used. For permeability experiments, cells were bathed with solution containing (in mM) 140 NaCl and 10 HEPES, pH 7.4. NaCl was reduced to 14 mM or replaced with N-Methyl-D-glucamine chloride (NMDG-Cl), sodium methanesulfonate (NaMeS) or sodium isothiocyanate (NaSCN). The osmolarity in the low NaCl solution was adjusted with sucrose.

For inside-out recordings, the pipette solution contained (in mM) 140 (or 14) NaCl, 5 EGTA and 10 HEPES, pH 7.2 with NaOH. The patches were maintained in nominally 0 Ca^2+^ solution containing (in mM) 140 NaCl, 10 HEDTA or 5 EGTA and 10 HEPES, pH 7.2. For dose-response relations, the bath solution was the same as that used in the pipette for whole-cell recordings. For permeability experiments using the dilution method, the bath solution contained (in mM) 140 NaCl, 10 HEPES and 0.1 or 1 mM CaCl_2_ and NaCl was changed to 280, 70, 28, or 14 mM. The osmolarity in the low-NaCl solution was adjusted with sucrose. In some experiments, NaCl was replaced with NMDG-Cl, NaMeS, NaSCN, NaBr, NaNO_3_ or NaI. All chemicals, unless otherwise stated, were purchased from Sigma-Aldrich (Milano, Italy).

### 4.4. Data Analysis

Data analysis and figures were made with IgorPro software (Wavemetrics, Lake Oswego, OR, USA). Data are presented as the mean ± sem (standard error of the mean), with n indicating the number of cells or excides patches. The double exponential function used to fit the data in Figure 1D was I = A_1_ exp (−t/t_1_) + A_2_ exp (−t/t_2_), where t_1_ and t_2_ are the time constants. The normality of the data was tested with the Shapiro–Wilk test, while the homogeneity of variance was tested with Levene’s test. For normally distributed data, statistical significance was determined using paired unpaired *t*-tests or ANOVA as appropriate. When a statistically significant difference was determined with ANOVA, a post-hoc Tukey test or Dunnett test was performed to evaluate which data groups showed significant differences. For non-normally distributed data, we used the Wilcoxon–Mann–Whitney test. For multicomparison of the data we used Dunn–Hollander–Wolfe test after Kruskal–Wallis analysis. Data in Figure 2F were analysed using the methods developed by Crawford and Garthwaite [62] using R. *p* values < 0.05 were considered significant.

## Data Availability

The data that support the findings of this study are available from the corresponding author upon reasonable request.

## Supplementary Materials

The following are available online at https://www.mdpi.com/article/10.3390/ijms22168578/s1.

## References

[1] Pedemonte N., Galietta L.J.V. (2014). Structure and Function of TMEM16 Proteins (Anoctamins). Physiol. Rev..

[2] Whitlock J.M., Hartzell H.C. (2017). Anoctamins/TMEM16 Proteins: Chloride Channels Flirting with Lipids and Extracellular Vesicles. Annu. Rev. Physiol..

[3] Falzone M.E., Malvezzi M., Lee B.-C., Accardi A. (2018). Known Structures and Unknown Mechanisms of TMEM16 Scramblases and Channels. J. Gen. Physiol..

[4] Kalienkova V., Clerico Mosina V., Paulino C. (2021). The Groovy TMEM16 Family: Molecular Mechanisms of Lipid Scrambling and Ion Conduction. J. Mol. Biol..

[5] Caputo A., Caci E., Ferrera L., Pedemonte N., Barsanti C., Sondo E., Pfeffer U., Ravazzolo R., Zegarra-Moran O., Galietta L.J.V. (2008). TMEM16A, a Membrane Protein Associated with Calcium-Dependent Chloride Channel Activity. Science.

[6] Yang Y.D., Cho H., Koo J.Y., Tak M.H., Cho Y., Shim W.-S., Park S.P., Lee J., Lee B., Kim B.-M. (2008). TMEM16A Confers Receptor-Activated Calcium-Dependent Chloride Conductance. Nature.

[7] Schroeder B.C., Cheng T., Jan Y.N., Jan L.Y. (2008). Expression Cloning of TMEM16A as a Calcium-Activated Chloride Channel Subunit. Cell.

[8] Pifferi S., Dibattista M., Menini A. (2009). TMEM16B Induces Chloride Currents Activated by Calcium in Mammalian Cells. Pflüg. Arch. Eur. J. Physiol..

[9] Stephan A.B., Shum E.Y., Hirsh S., Cygnar K.D., Reisert J., Zhao H. (2009). ANO2 Is the Cilial Calcium-Activated Chloride Channel That May Mediate Olfactory Amplification. Proc. Natl. Acad. Sci. USA.

[10] Benedetto R., Cabrita I., Schreiber R., Kunzelmann K. (2018). TMEM16A Is Indispensable for Basal Mucus Secretion in Airways and Intestine. FASEB J..

[11] Pietra G., Dibattista M., Menini A., Reisert J., Boccaccio A. (2016). The Ca^2+^-Activated Cl^−^ Channel TMEM16B Regulates Action Potential Firing and Axonal Targeting in Olfactory Sensory Neurons. J. Gen. Physiol..

[12] Dibattista M., Pifferi S., Boccaccio A., Menini A., Reisert J. (2017). The Long Tale of the Calcium Activated Cl- Channels in Olfactory Transduction. Channels Austin Tex..

[13] Amjad A., Hernandez-Clavijo A., Pifferi S., Maurya D.K., Boccaccio A., Franzot J., Rock J., Menini A. (2015). Conditional Knockout of TMEM16A/Anoctamin1 Abolishes the Calcium-Activated Chloride Current in Mouse Vomeronasal Sensory Neurons. J. Gen. Physiol..

[14] Leblanc N., Forrest A.S., Ayon R.J., Wiwchar M., Angermann J.E., Pritchard H.A.T., Singer C.A., Valencik M.L., Britton F., Greenwood I.A. (2015). Molecular and Functional Significance of Ca^2+^-Activated Cl^−^ Channels in Pulmonary Arterial Smooth Muscle. Pulm. Circ..

[15] Suzuki J., Fujii T., Imao T., Ishihara K., Kuba H., Nagata S. (2013). Calcium-Dependent Phospholipid Scramblase Activity of TMEM16 Protein Family Members. J. Biol. Chem..

[16] Suzuki J., Umeda M., Sims P.J., Nagata S. (2010). Calcium-Dependent Phospholipid Scrambling by TMEM16F. Nature.

[17] Bevers E.M., Williamson P.L. (2016). Getting to the Outer Leaflet: Physiology of Phosphatidylserine Exposure at the Plasma Membrane. Physiol. Rev..

[18] Castoldi E., Collins P.W., Williamson P.L., Bevers E.M. (2011). Compound Heterozygosity for 2 Novel TMEM16F Mutations in a Patient with Scott Syndrome. Blood.

[19] Ehlen H.W.A., Chinenkova M., Moser M., Munter H.-M., Krause Y., Gross S., Brachvogel B., Wuelling M., Kornak U., Vortkamp A. (2013). Inactivation of Anoctamin-6/Tmem16f, a Regulator of Phosphatidylserine Scrambling in Osteoblasts, Leads to Decreased Mineral Deposition in Skeletal Tissues. J. Bone Miner. Res..

[20] Batti L., Sundukova M., Murana E., Pimpinella S., De Castro Reis F., Pagani F., Wang H., Pellegrino E., Perlas E., Di Angelantonio S. (2016). TMEM16F Regulates Spinal Microglial Function in Neuropathic Pain States. Cell Rep..

[21] Zaitseva E., Zaitsev E., Melikov K., Arakelyan A., Marin M., Villasmil R., Margolis L.B., Melikyan G.B., Chernomordik L.V. (2017). Fusion Stage of HIV-1 Entry Depends on Virus-Induced Cell Surface Exposure of Phosphatidylserine. Cell Host Microbe.

[22] Braga L., Ali H., Secco I., Chiavacci E., Neves G., Goldhill D., Penn R., Jimenez-Guardeño J.M., Ortega-Prieto A.M., Bussani R. (2021). Drugs That Inhibit TMEM16 Proteins Block SARS-CoV-2 Spike-Induced Syncytia. Nature.

[23] Yang H., Kim A., David T., Palmer D., Jin T., Tien J., Huang F., Cheng T., Coughlin S.R., Jan Y.N. (2012). TMEM16F Forms a Ca^2+^-Activated Cation Channel Required for Lipid Scrambling in Platelets during Blood Coagulation. Cell.

[24] Kunzelmann K., Nilius B., Owsianik G., Schreiber R., Ousingsawat J., Sirianant L., Wanitchakool P., Bevers E.M., Heemskerk J.W.M. (2014). Molecular Functions of Anoctamin 6 (TMEM16F): A Chloride Channel, Cation Channel, or Phospholipid Scramblase?. Pflug. Arch..

[25] Picollo A., Malvezzi M., Accardi A. (2015). TMEM16 Proteins: Unknown Structure and Confusing Functions. J. Mol. Biol..

[26] Shimizu T., Iehara T., Sato K., Fujii T., Sakai H., Okada Y. (2013). TMEM16F Is a Component of a Ca^2+^-Activated Cl^−^ Channel but Not a Volume-Sensitive Outwardly Rectifying Cl^−^ Channel. Am. J. Physiol. Cell Physiol..

[27] Grubb S., Poulsen K.A., Juul C.A., Kyed T., Klausen T.K., Larsen E.H., Hoffmann E.K. (2013). TMEM16F (Anoctamin 6), an Anion Channel of Delayed Ca^2+^ Activation. J. Gen. Physiol..

[28] Henkel B., Drose D.R., Ackels T., Oberland S., Spehr M., Neuhaus E.M. (2015). Co-Expression of Anoctamins in Cilia of Olfactory Sensory Neurons. Chem. Senses.

[29] Ousingsawat J., Wanitchakool P., Kmit A., Romao A.M., Jantarajit W., Schreiber R., Kunzelmann K. (2015). Anoctamin 6 Mediates Effects Essential for Innate Immunity Downstream of P2X7 Receptors in Macrophages. Nat. Commun..

[30] Aoun J., Hayashi M., Sheikh I.A., Sarkar P., Saha T., Ghosh P., Bhowmick R., Ghosh D., Chatterjee T., Chakrabarti P. (2016). Anoctamin 6 Contributes to Cl- Secretion in Accessory Cholera Enterotoxin (Ace)-Stimulated Diarrhea: An essential role for phosphatidylinositol 4,5-bisphosphate (PIP2) signaling in cholera. J. Biol. Chem..

[31] Muratori C., Pakhomov A.G., Gianulis E., Meads J., Casciola M., Mollica P.A., Pakhomova O.N. (2017). Activation of the Phospholipid Scramblase TMEM16F by Nanosecond Pulsed Electric Fields (NsPEF) Facilitates Its Diverse Cytophysiological Effects. J. Biol. Chem..

[32] Simões F., Ousingsawat J., Wanitchakool P., Fonseca A., Cabrita I., Benedetto R., Schreiber R., Kunzelmann K. (2018). CFTR Supports Cell Death through ROS-Dependent Activation of TMEM16F (Anoctamin 6). Pflug. Arch..

[33] Kim H.J., Jun I., Yoon J.S., Jung J., Kim Y.K., Kim W.K., Kim B.J., Song J., Kim S.J., Nam J.H. (2015). Selective Serotonin Reuptake Inhibitors Facilitate ANO6 (TMEM16F) Current Activation and Phosphatidylserine Exposure. Pflug. Arch..

[34] Alvadia C., Lim N.K., Clerico Mosina V., Oostergetel G.T., Dutzler R., Paulino C. (2019). Cryo-EM Structures and Functional Characterization of the Murine Lipid Scramblase TMEM16F. eLife.

[35] Nguyen D.M., Chen L.S., Yu W.-P., Chen T.-Y. (2019). Comparison of Ion Transport Determinants between a TMEM16 Chloride Channel and Phospholipid Scramblase. J. Gen. Physiol..

[36] Ye W., Han T.W., He M., Jan Y.N., Jan L.Y. (2019). Dynamic Change of Electrostatic Field in TMEM16F Permeation Pathway Shifts Its Ion Selectivity. eLife.

[37] Betto G., Cherian O.L., Pifferi S., Cenedese V., Boccaccio A., Menini A. (2014). Interactions between Permeation and Gating in the TMEM16B/Anoctamin2 Calcium-Activated Chloride Channel. J. Gen. Physiol..

[38] Cenedese V., Betto G., Celsi F., Cherian O.L., Pifferi S., Menini A. (2012). The Voltage Dependence of the TMEM16B/Anoctamin2 Calcium-Activated Chloride Channel Is Modified by Mutations in the First Putative Intracellular Loop. J. Gen. Physiol..

[39] Barry P.H. (2006). The Reliability of Relative Anion-Cation Permeabilities Deduced from Reversal (Dilution) Potential Measurements in Ion Channel Studies. Cell Biochem. Biophys..

[40] Le T., Jia Z., Le S.C., Zhang Y., Chen J., Yang H. (2019). An Inner Activation Gate Controls TMEM16F Phospholipid Scrambling. Nat. Commun..

[41] Ye W., Han T.W., Nassar L.M., Zubia M., Jan Y.N., Jan L.Y. (2018). Phosphatidylinositol-(4, 5)-Bisphosphate Regulates Calcium Gating of Small-Conductance Cation Channel TMEM16F. Proc. Natl. Acad. Sci. USA.

[42] Yu K., Duran C., Qu Z., Cui Y.-Y., Hartzell H.C. (2012). Explaining Calcium-Dependent Gating of Anoctamin-1 Chloride Channels Requires a Revised Topology. Circ. Res..

[43] Yu K., Whitlock J.M., Lee K., Ortlund E.A., Cui Y.Y., Hartzell H.C. (2015). Identification of a Lipid Scrambling Domain in ANO6/TMEM16F. eLife.

[44] Di Zanni E., Gradogna A., Scholz-Starke J., Boccaccio A. (2018). Gain of Function of TMEM16E/ANO5 Scrambling Activity Caused by a Mutation Associated with Gnathodiaphyseal Dysplasia. Cell. Mol. Life Sci. CMLS.

[45] Whitlock J.M., Yu K., Cui Y.Y., Hartzell H.C. (2018). Anoctamin 5/TMEM16E Facilitates Muscle Precursor Cell Fusion. J. Gen. Physiol..

[46] Lin H., Roh J., Woo J.H., Kim S.J., Nam J.H. (2018). TMEM16F/ANO6, a Ca^2+^-Activated Anion Channel, Is Negatively Regulated by the Actin Cytoskeleton and Intracellular MgATP. Biochem. Biophys. Res. Commun..

[47] Scudieri P., Caci E., Venturini A., Sondo E., Pianigiani G., Marchetti C., Ravazzolo R., Pagani F., Galietta L.J.V. (2015). Ion Channel and Lipid Scramblase Activity Associated with Expression of TMEM16F/ANO6 Isoforms. J. Physiol..

[48] Fakler B., Adelman J.P. (2008). Control of K(Ca) Channels by Calcium Nano/Microdomains. Neuron.

[49] Takeuchi H., Kurahashi T. (2018). Second Messenger Molecules Have a Limited Spread in Olfactory Cilia. J. Gen. Physiol..

[50] Adomaviciene A., Smith K.J., Garnett H., Tammaro P. (2013). Putative Pore-Loops of TMEM16/Anoctamin Channels Affect Channel Density in Cell Membranes. J. Physiol..

[51] Jiang T., Yu K., Hartzell H.C., Tajkhorshid E. (2017). Lipids and Ions Traverse the Membrane by the Same Physical Pathway in the NhTMEM16 Scramblase. eLife.

[52] Sachs F. (2010). Stretch-Activated Ion Channels: What Are They?. Physiology.

[53] Pathak M.M., Tran T., Hong L., Joós B., Morris C.E., Tombola F. (2016). The Hv1 Proton Channel Responds to Mechanical Stimuli. J. Gen. Physiol..

[54] Brohawn S.G., Su Z., MacKinnon R. (2014). Mechanosensitivity Is Mediated Directly by the Lipid Membrane in TRAAK and TREK1 K+ Channels. Proc. Natl. Acad. Sci. USA.

[55] Yang T., Colecraft H.M. (2015). Calmodulin Regulation of TMEM16A and 16B Ca2+-Activated Chloride Channels. Channels.

[56] Jung J., Nam J.H., Park H.W., Oh U., Yoon J.-H., Lee M.G. (2013). Dynamic Modulation of ANO1/TMEM16A HCO3(-) Permeability by Ca^2+^/Calmodulin. Proc. Natl. Acad. Sci. USA.

[57] Yu Y., Chen T.-Y. (2015). Purified Human Brain Calmodulin Does Not Alter the Bicarbonate Permeability of the ANO1/TMEM16A Channel. J. Gen. Physiol..

[58] Huang F., Wang X., Ostertag E.M., Nuwal T., Huang B., Jan Y.-N., Basbaum A.I., Jan L.Y. (2013). TMEM16C Facilitates Na^+^-Activated K^+^ Currents in Rat Sensory Neurons and Regulates Pain Processing. Nat. Neurosci..

[59] Pan B., Akyuz N., Liu X.-P., Asai Y., Nist-Lund C., Kurima K., Derfler B.H., György B., Limapichat W., Walujkar S. (2018). TMC1 Forms the Pore of Mechanosensory Transduction Channels in Vertebrate Inner Ear Hair Cells. Neuron.

[60] Ávalos Prado P., Häfner S., Comoglio Y., Wdziekonski B., Duranton C., Attali B., Barhanin J., Sandoz G. (2021). KCNE1 Is an Auxiliary Subunit of Two Distinct Ion Channel Superfamilies. Cell.

[61] Dulin N.O. (2020). Calcium-Activated Chloride Channel ANO1/TMEM16A: Regulation of Expression and Signaling. Front. Physiol..

[62] Crawford J.R., Garthwaite P.H. (2007). Using Regression Equations Built from Summary Data in the Neuropsychological Assessment of the Individual Case. Neuropsychology.

