# Supplementary material for: Anion and Cation Permeability of the Mouse TMEM16F Calcium-Activated Channel
Source: Int J Mol Sci. 2021 Aug 9;22(16):8578. doi: 10.3390/ijms22168578 (PMC8395294; doi:10.3390/ijms22168578)
Supplement: Supplementary file 1 [file ijms-22-08578-s001.zip › ijms-1309263-supplementary.pdf]

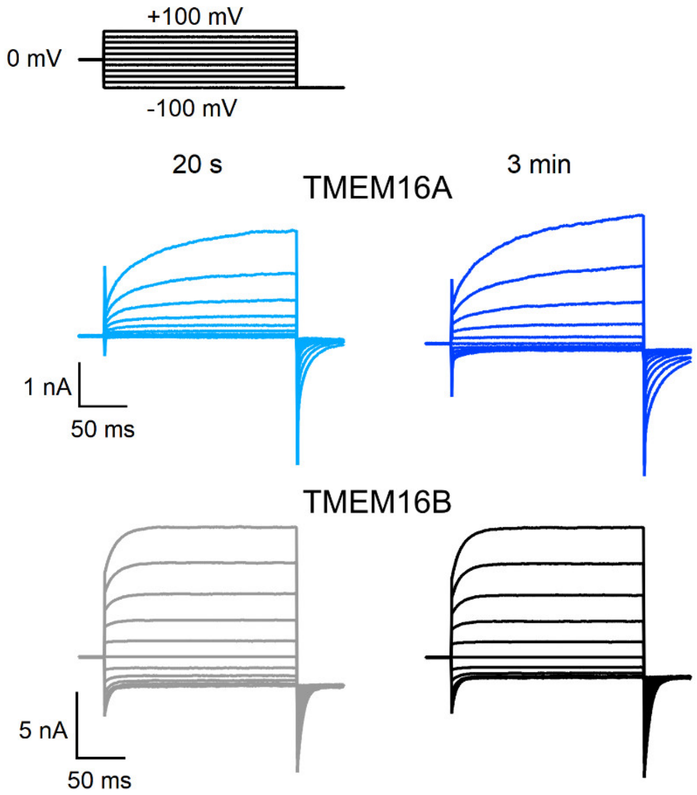

Figure S1. Kinetics of Ca^{2+}-dependent activation of the TMEM16A and TMEM16B-mediated currents. Whole-cell recordings obtained from cells transfected with TMEM16A (upper traces) or TMEM16B with a pipette solution containing 0.36 μM Ca^{2+} (TMEM16A) or 1.5 μM Ca^{2+} (TMEM16B). The recordings were obtained 20 s or 3 min after membrane breaking, as indicated. The voltage protocol is shown at the top.

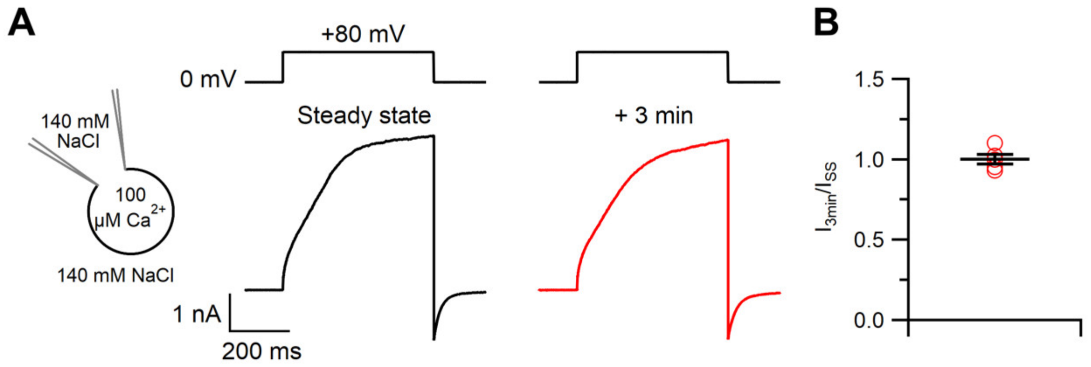

Figure S2. Absence of rundown of TMEM16F-mediated current in whole-cell recordings after reaching the steady state level. (**A**) Representative whole-cell voltage-clamp recordings obtained from TMEM16F wt-transfected cells with intracellular solution containing 100 μM Ca^{2+}. Voltage protocols are shown at the top of the panels. The black trace is the steady state current measured some minutes after membrane breaking (Fig 1. A-C). The red trace is the current measured after 3 additional minutes. (**B**) Ratios of the current measured 3 min after reaching the steady state and the current at steady state from several cells (n=5).

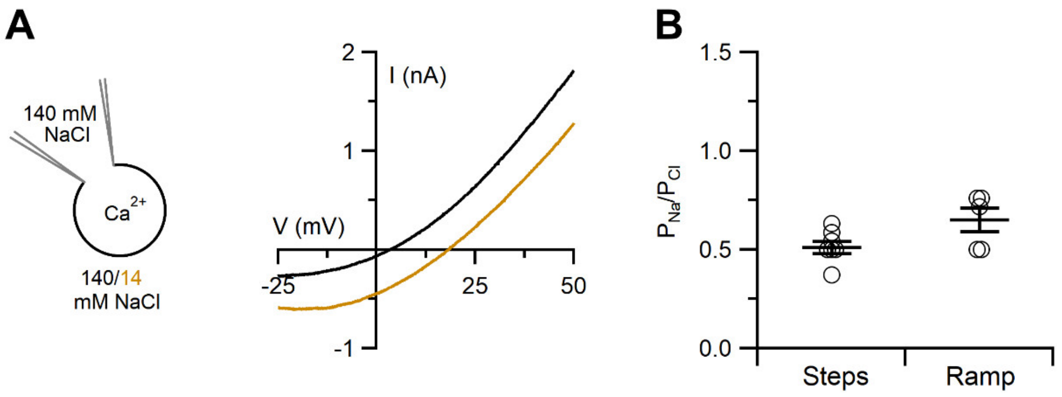

Figure S3. TMEM16F wt ion permeability ratio P_{Na}/P_{Cl} in the whole-cell configuration measured with voltage ramp. (**A**) Representative whole-cell voltage-clamp recordings obtained from TMEM16F wt-transfected cells with intracellular solution containing 50 μM Ca^{2+}. A cell was first exposed to a control solution containing 140 mM NaCl, then to 14 mM NaCl. Current was activated by a voltage step to +100 mV for 1 s and then a ramp from +80 to -80 mV at 0.36 mV/ms was applied. (**B**) Comparison of P_{Na}/P_{Cl} calculated with the Goldman-Hodgkin-Katz equation from reversal potentials measured with voltage steps (as in Figure 3A-C, F) or with voltage ramps (n = 5-7; p>0.05 t-test).

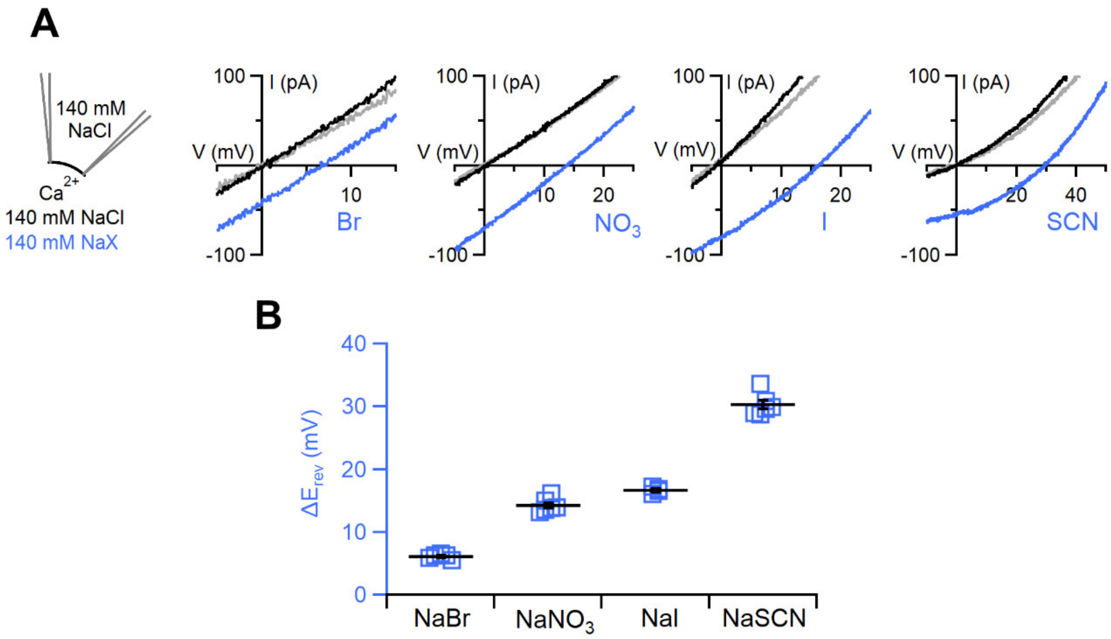

Figure S4. Anion permeability of TMEM16F wt in inside-out patches. **(A)** One inside-out patch expressing TMEM16F wt was exposed to bath solutions containing 140 mM NaCl (black traces) or the Na salt of other anions (blue traces), as indicated. The gray traces represent the wash out with NaCl. IV relations were determined by voltage ramps from +100 to -80 mV. Only regions around the reversal potentials are shown. Currents were activated by 1 mM $CaCl_2$ and leakage currents measured in 0 Ca^{2+} were subtracted. (**B**) Scatter dot plots with average ± sem of shift of reversal potentials after the substitution with the indicated salts (n = 4-6).

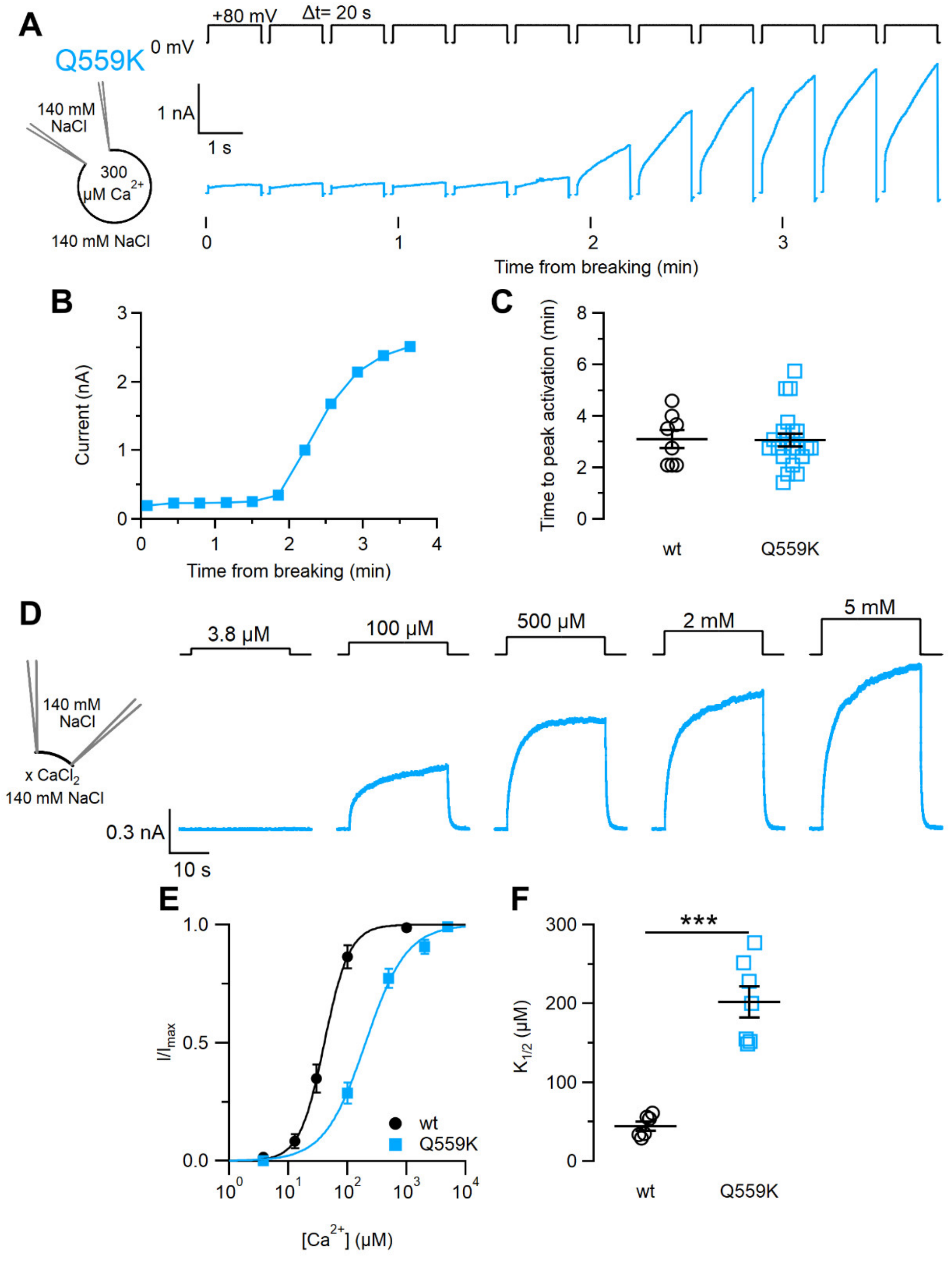

Figure S5. Kinetics of Ca^{2+}-dependent activation and Ca^{2+} sensitivity of TMEM16F Q559K mutant. (**A**) Whole-cell recordings obtained from TMEM16F Q559K mutant-transfected cells with a pipette solution containing 300 μM Ca^{2+}. The development of the current with time was monitored by applying a +80 mV voltage step every 20 s, as indicated in the upper traces. (**B**) Current amplitudes at the end of voltage steps were plotted against the time after membrane breaking for the recordings in (A). (**C**) Comparison of the time required to reach the maximal current in TMEM16F wt or Q559K mutant-transfected cells (n = 8-21). (**D**) An inside-out excised membrane patch expressing TMEM16F Q559K mutant was stimulated with solutions containing different free Ca^{2+} concentrations at the time indicated in the upper traces. The holding potential was +60 mV. (**E**) Dose–response relations of activation by Ca^{2+} obtained by normalized currents fitted to the Hill equation. (F) Comparison of $K_{1/2}$ obtained from TMEM16F wt and Q559K mutant channels (n = 7, ***$p < 0.001$ unpaired t-test).

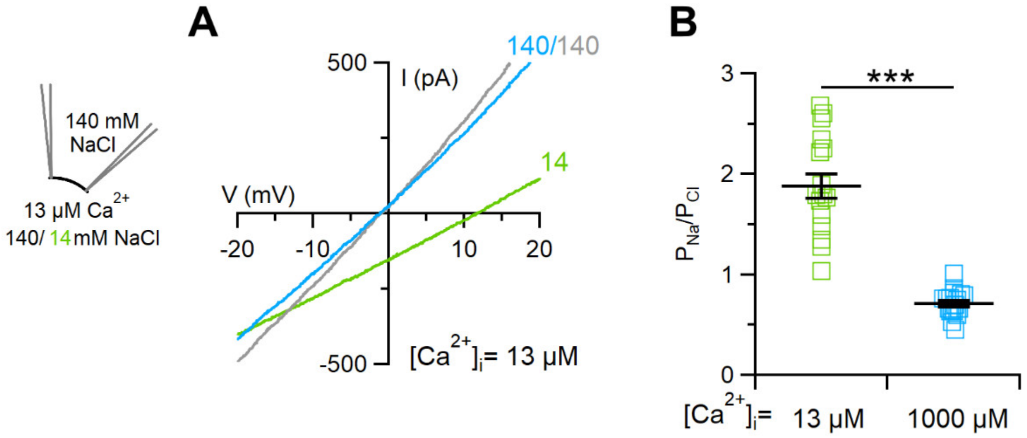

Figure S6. TMEM16F Q559K ion permeability ratio P_{Na}/P_{Cl} in excised inside-out patches depends on intracellular Ca^{2+} concentration. (**A**) An inside-out patch expressing TMEM16F Q559K mutant was exposed to 140 or 14 mM cytoplasmatic NaCl. IV relations were determined by voltage ramps from +100 to -80 mV. Only the regions around the reversal potentials are shown. Currents were activated by 13 µM Ca^{2+} and leakage currents measured in 0 Ca^{2+} were subtracted. (**B**) Comparison of P_{Na}/P_{Cl} for currents activated by 13 µM or 1 mM Ca^{2+} (data in 1 mM Ca^{2+} are from Figure 6F; n = 16-18, ***$p < 0.001$, $p = 2.12 \times 10^{-11}$ unpaired t-test).
